# Sense of Coherence and Physical Health. A “Copenhagen Interpretation” of Antonovsky's SOC Concept

**DOI:** 10.1100/tsw.2008.59

**Published:** 2008-04-20

**Authors:** P. Christian Endler, Thomas M. Haug, Heinz Spranger

**Affiliations:** Interuniversity College for Health and Development Graz, Castle of Seggau, Austria

**Keywords:** salutogenesis, sense of coherence, physical health, psychological health

## Abstract

According to Antonovsky's (Aaron Antonovsky, 1923–1994) sense of coherence (SOC) model, persons with a high SOC have the ability to benefit from their general defense mechanisms in order to overcome stressful situations. In a health-disease continuum, this leads to the development towards health. However, Antonovsky's global hypothesis that the strength of the SOC may influence the physical health status of a person could not be proven.

Flensborg-Madsen et al. from Copenhagen were able to provide a new access regarding SOC and health. They investigated the mixture of emotional aspects and mental constructions as a possible cause for fairly low correlation between SOC and physical health. Thus, in an empirical way, they described “emotional coherence” in relation to physical health, while “mental coherence” was linked to *psychological* health. These authors introduced the idea of applying a shortened version of the original 29-item SOC questionnaire, but have not yet developed or tested the shortened questionnaire. Backed by their important findings, it appears to be promising to consider the use of the SOC questionnaire as standardized by Antonovsky, but cleared of the items regarding “predictability”, i.e., Flensborg-Madsen et al. suggested that the items on “predictability” be excluded from the SOC scale when a correlation to physical health is to be investigated. Further investigations in this area of research will be of high impact, not only for health sciences, but also for medical practice.

## INTRODUCTION

In modern health sciences, the "health concept" is strongly influenced by salutogenesis, founded in the 1970s by the American-Israeli medical sociologist Aaron Antonovsky (1923–1994)[1]. According to this model, health and disease represent a health-disease continuum, rather than opposing conditions. The individual sense of coherence (SOC) has a high impact on the positioning of the continuum.

The core message of the SOC model is based on the assumption that persons with a high SOC have the ability to benefit from their general defense mechanisms in order to overcome stressful situations. In a health-disease continuum, this leads to the development towards health and respectively supports health maintenance. The SOC scale contains aspects of comprehensibility, manageability, and meaningfulness, demonstrating a coping strategy for different requirements of everyday life[1]. To measure the SOC, Antonovsky developed questionnaires, which were translated into 33 languages and used in 32 countries[2]. Further, Antonovsky's hypothesis included the assumption that the strength of the SOC may have direct physiological consequences and, hence, may influence the health status of a person[1].

Since that time, the SOC concept has increased in popularity and impact for health, medical, psychological, and sociological sciences[3]. Nevertheless, the concept has generated numerous critical remarks[4]: The lack of empirical replicability of the subscale structure within the SOC scale (predictability, manageability, meaningfulness) has to be mentioned in the first place. In fact, in order to be of any practical use, the SOC value has to be understood as a total value in terms of a one-dimensional construction[1]. One of the main criticisms is the high (negative) correlation between the SOC scale and the scales measuring anxiety, depression, and neuroticism[4], which has made the independence (self-reliance) of the SOC scale questionable. Antonovsky's hypothesis that a higher SOC would correlate with better physiological health has not been proven. Accordant correlations in most of the cases are not sufficiently distinct[4].

Trine Flensborg-Madsen and collaborators in Copenhagen[4,5,6,7,8,9,10,11] were able to provide an important access regarding the correlation of SOC and physical health.

## METHODS AND RESULTS

Antonovsky's standardization of the SOC items is considered a problem by the Copenhagen authors. Regarding the whole SOC concept, the concept of "predictability" appears to be over-rated and over-represented in the operationalization of the questions. The Copenhagen authors suggest that the SOC subscales on comprehensibility and manageability be cleared – and thus the SOC – by sorting out the aspect of predictability[7,8]. (In the paper presented here, we cannot discuss whether this reassessment of the SOC would improve the original Antonovsky concept.)

We would like to point out the working hypothesis of Flensborg-Madsen et al., i.e., that by clarifying the SOC questionnaire regarding the aspect of "predictability", a correlation with physical health could be determined. In other words, this would provide a tool to observe the link between emotional coherence and physical health.

Flensborg-Madsen et al. introduced their idea of applying a shortened version of the original 29-item SOC questionnaire, but have not yet developed or tested the shortened questionnaire. Rather, they developed their own SOC scale with alternative items (SOC-E and SOC-II), which showed a direct correlation with physical health[7,8,9,10,11], when empirically tested. Backed by these important findings, it appears to be promising to consider their first idea of using the SOC questionnaire as standardized by Antonovsky, but cleared of the items regarding "predictability".

## DISCUSSION

It remains to be discussed whether the reassessment of the SOC questionnaire really would improve the original Antonovsky SOC concept.

Flensborg-Madsen et al. suggested a basis for analysis and exploration of the correlation between aspects of the SOC and physical health. It will be up to health scientists to prove their concept. The option to differentiate between emotional coherence ("manageability" and "meaningfulness") and mental coherence ("predictability"), and further to abstain from "predictability", demonstrates a promising method to disclose consistently Antonovsky's hypothesis of correlation between SOC and physical health. An integrative application of this model would have a high impact for medical practice and its use would no longer be restricted to health promotion only.
